# Baseline correlates of functional impairment at 12 months in young people with borderline personality disorder: findings from the MOBY trial

**DOI:** 10.1007/s00787-025-02742-5

**Published:** 2025-05-28

**Authors:** Andrew M. Chanen, Holly E Andrewes, Katie Nicol, Henry J Jackson, Sue M. Cotton, John Gleeson, Christopher G. Davey, Louise McCutcheon, Sharnel Perera, Victoria Rayner, Jennifer K. Betts

**Affiliations:** 1 Orygen, 35 Poplar Rd, Parkville, VIC 3052 Australia; 2 Centre for Youth Mental Health, The University of Melbourne, 35 Poplar Rd, Parkville, VIC 3052 Australia; 3 Melbourne School of Psychological Sciences, The University of Melbourne, Royal Parade, Parkville, VIC 3010 Australia; 4 Turner Institute of Brain and Mental Health, School of Psychological Sciences, Monash University, Level 5, 18 Innovation Walk Clayton Campus, Clayton, VIC 3800 Australia; 5 Healthy Brain and Mind Research Centre, School of Behavioural and Health Sciences, Australian Catholic University, The Daniel Mannix Building, Level 5, 17-29 Young St, Fitzroy, 3065 Australia; 6 Department of Psychiatry, The University of Melbourne, Grattan Street, Parkville, 3010 Australia

**Keywords:** Psychiatry, Youth, Adolescence, Employment, Early intervention, Functioning

## Abstract

No studies have investigated baseline predictors of functioning among young people receiving treatment for BPD. The current study aimed to identify the baseline demographic, psychopathological and treatment correlates of interpersonal problems and social adjustment at 12-month follow-up among young people with BPD, who received early intervention in the Monitoring Outcomes of BPD in Youth (MOBY) randomised controlled trial. Of 139 randomised young people (aged 15 to 25 years) who completed interview and self-report measures at baseline (pre-treatment), 98 (M_age_ = 18.7, *SD* = 2.7) provided data on the same measures at 12 months (primary endpoint). Adjusting for other demographic, clinical and treatment characteristics, hierarchical regression analyses revealed that fewer interpersonal problems at 12 months were predicted by fewer interpersonal problems (β = 0.41, *p* = 0.02), fewer co-occurring personality disorder diagnoses (β = -0.31, *p* = 0.04), and lower BPD severity (β = -0.31, *p* = 0.04) at baseline (*F*^2^ = 0.52). Higher social adjustment score at 12 months was predicted by having at least one caregiver engaged in employment at baseline (β = -0.25, *p* = 0.04, *F*^2^ = 0.41). These findings suggest that young people presenting with lower severity of BPD, fewer interpersonal problems at baseline, and at least one caregiver in employment are more likely to have better functional outcomes following early intervention. Suicide attempts (SA) and self-harm (SH) were not found to be predictors of functional outcome, suggesting that early intervention programs reconsider the disproportionate influence of SA and SH upon decisions about entry or exclusion into treatment programs or clinical trials. Future research should focus upon individual or household indicators of social disadvantage as moderators of outcome in early intervention for BPD.

**Supplementary Information:**

The online version contains supplementary material available at 10.1007/s00787-025-02742-5.

## Introduction

Borderline personality disorder (BPD) usually emerges during the developmental period spanning from puberty to emerging adulthood, usually referred to as ‘young people’ or ‘transition age youth’ [1, 2]. BPD is associated with significant psychosocial functional impairment that persists long after its diagnostic features have attenuated [3]. Such impairments in the capacity for adaptive and age-appropriate functioning in intimate, social, occupational and educational environments [4, 5] emerge early in the course of BPD and have the potential to disrupt the acquisition of essential skills for adult role functioning [6].

Among both community dwelling adolescents [7] and young people aged 16–24 years with their first presentation to clinical services for BPD care [8], functional problems include fewer and shorter friendships and higher odds of school or work-related problems [7], or unemployment [8], compared with other adolescents or young people with other mental disorders. Among an epidemiological sample of community dwelling 24 year-olds, higher BPD severity has been associated with a higher likelihood of smoking cigarettes, experiencing anxiety or depression, being in receipt of welfare payments, and a lower likelihood of gaining tertiary qualifications [9]. In support of this latter finding, approximately 40% of young people (aged 15–25 years) recently diagnosed with BPD and presenting for the first time for BPD treatment were found to be disengaged from education, employment and training [10].

Early intervention aims to prevent or ameliorate these functional impairments, before they become entrenched [11]. Yet, only four randomised controlled trials (RCTs) of structured psychological interventions (versus active comparators) for young people with BPD have measured psychosocial functioning as an outcome [12–15], and only one of these nominated psychosocial functioning as the primary outcome [15]. All four RCTs reported slight to moderate improvements in psychosocial functioning, with only one of these finding superiority of the treatment over the comparator [13].

To the authors’ knowledge, the demographic, clinical, or specific treatment characteristics that might predict functional outcomes among this population are still unknown. Addressing this gap might offer insight into the individual characteristics of young people who experience higher levels of psychosocial functioning following treatment. Possible correlates of psychosocial functioning in this age group can only be gleaned from naturalistic prospective studies, which have found that lower BPD criteria scores are associated with fewer functional problems at two- [16], four- [17] and twenty-year [18] follow-up. Furthermore, lower personality disorder severity among a community sample of 24 year-olds (*n* = 1443) has been associated with higher odds of experiencing a long-term relationship at age 35 years, when adjusting for other pre-existing mental health issues, substance or social problems [9].

Studies among adults with BPD provide limited guidance for selecting potential correlates of psychosocial functioning. Meta-analytic data from naturalistic and treatment research investigating longitudinal functional outcomes at 5-year follow-up found that being male was correlated with a greater improvement in functioning, while no relationship was found for other demographic characteristics, such as age [3]. Only four RCTs of treatments for adults with BPD have investigated the individual and treatment characteristics that might correlate with functional outcomes [19–22]. Three of these have focused upon process measures (e.g., emotion dysregulation, post-traumatic stress disorder (PTSD) cognitions and PTSD severity), rather than baseline characteristics [19–21]. Data from an RCT of Systems Training for Emotional Predictability and Problem Solving versus Treatment as Usual (*N* = 164) found that baseline global assessment scale (GAS) score was positively associated with global functioning at 12-month follow-up, regardless of treatment group [22].

The current study aimed to investigate baseline correlates of higher psychosocial functioning at 12-month follow-up among young people with ‘first-presentation’ BPD who had received early intervention for BPD. Psychosocial functioning was defined as a young person’s ability to develop and maintain interpersonal relationships and to perform in an age-appropriate manner in work, education and social and leisure activities. Given the limited number of studies and heterogeneity of the above findings, this exploratory analysis specifically assessed for inclusion variables found to be associated with better functional outcomes in longitudinal studies and RCTs among adults with BPD, viz. BPD severity, sex, PTSD diagnoses, emotion dysregulation and global functioning.

## Method

### Design

The present study involved a secondary and exploratory analysis of the Monitoring Outcomes of BPD in Youth (MOBY) RCT, details of which have been published elsewhere [15, 23]. Briefly, young people with first-presentation BPD were randomised to one of three early intervention treatments for BPD and assessed at baseline and then at 3, 6, 12 and 18 months. The primary outcome was psychosocial functioning at the 12-month post-randomisation primary endpoint. Consequently, the 12-month end-point was used in the current study. This was jointly measured using the Social Adjustment Scale Self-report and the Inventory of Interpersonal Problems Circumplex Version. As treatment group allocation was found not to have a significant effect upon these outcomes [15], the three treatment groups were combined for the current analyses.

### Participants

Ninety-eight participants who had completed the 12-month outcome measures were included in the current study. Upon entry to the study, participants were aged 15 to 25 years, had recently diagnosed BPD and had never received evidence-based BPD treatment (i.e., first-presentation BPD).

Key exclusion criteria were (1) DSM-IV psychotic disorder within the past 12 months; (2) lifetime diagnosis of a schizophrenia spectrum or bipolar I or II disorder; and (3) prior evidence-based treatment for BPD. All participants (and a parent/legal guardian for minors) gave written informed consent. The study was approved by the Melbourne Health Human Research Ethics Committee (HREC2010.055).

### Measures

Interpersonal problems were measured withthe 64-item, self-report Inventory of Interpersonal Problems Circumplex Version [24]. Scale scores range from 0 to 264. Lower scores on this scale indicate fewer interpersonal problems. Social adjustment was measured with the 54-item, self-report Social Adjustment Scale-Self Report [SAS-SR; 25], which rates functioning over the previous two weeks in three main domains: work, social and leisure activities, and relationships. Lower scores on this scale indicate higher levels of social adjustment.

Demographic variables collected at baseline are listed in Table 1. Social disadvantage was measured using the Australian Bureau of Statistics Index of Relative Social Disadvantage and was divided into tertiles (Low, Medium, High).

DSM diagnoses were derived using the Structured Clinical Interview for DSM-IV Axis I – Patient Edition [SCID-I/P; 26] and SCID-II [27]. In keeping with previous practice, criterion B for Antisocial personality disorder was ignored for participants aged under 18 years [15].

BPD severity was derived from the BPD severity index (BPDSI-IV), which assesses for the nine DSM-IV-TR BPD criteria (identical to DSM-5) over the previous 3 months [28]. Total scores range from 0 to 90.

Emotion regulation was measured via the 36-item, self-report Difficulties in Emotion Regulation Scale [DERS; 29], with higher scores indicating higher levels of emotion dysregulation.

Other measures included the 10-item Montgomery–Åsberg Depression Rating Scale [MADRS; 30], the clinician-rated Social and Occupational Functioning Assessment Scale [SOFAS; 31]. The classes and number of substances used over the past month were identified via the Opiate Treatment Index [OTI; 32]. The total number and classes of medications was assessed with a questionnaire which identified all medications used by participants at baseline. Client satisfaction with the service was assessed via an 8-item questionnaire [33]. Satisfaction scores were averaged across all time points (baseline, 3, 6, and 12 months) to provide a final score.

Several other variables (including self-harm and suicide attempts) were identified, but eliminated, prior to the final regression analysis due to multicollinearity with other independent variables or insufficient correlation with the dependent variables (see Supplementary Tables 1 and statistical analysis section for details).

### Statistical analyses

Two hierarchical multiple regression models were conducted to determine the symptom, behavioural (measured at baseline) and treatment characteristics (measured across a 12-month period) that predict social adjustment (Block 1; see Table 2) or interpersonal problems (Block 2; see Table 3) at 12 months. Demographic characteristics were entered into the model first (Block 1a, 2a), followed by symptom/behavioural characteristics (Block 1b, 2b) and finally treatment characteristics (Block 1c, 2c) to assess whether the addition of each group of variables explained a significant variation in the dependent variable. Preliminary analyses were completed to ensure all assumptions were met.

Due to the dearth of research investigating the relationship between symptomatic, behavioural and treatment characteristics and functional outcomes in longitudinal studies and RCTs, this research explored a range of potential predictors of functional outcomes. To reduce the number of dependent variables entered into the final models, variables were removed if there was an insufficient response rate (less than 10%), dichotomous variables were removed where similar continuous variables existed, and overlapping numeric variables were recoded into one variable or the least relevant variable was removed. A correlation analysis (Pearsons or Point Biserial) was then completed to identify multicollinearity between independent variables, with duplicate variables removed if their correlation was > 0.7 [34]. Variables not correlated with the independent variables at a significance level of *p* < 0.15 were also removed (see Supplementary Table 1). All assumptions relating to hierarchical regression were assessed for each model [34]. From these models, variance (R²), change in variance (ΔR²) and the F statistic (ΔF) are presented along with the standardised regression values (𝛽) and squared semi partial values (sri^2^). Cohen’s f^2^ was used to identify effect sizes with f^2^ ≥ 0.02, f^2^ ≥ 0.15, and f^2^ ≥ 0.35 representing small, medium, and large effect sizes, respectively [35]. All statistical analyses were completed using IBM^®^ SPSS^®^ Statistics 27.

## Results

### Participants

Table 1 shows that the majority of participants were female, identified as medium or high social disadvantage status, and had a co-occurring mood or anxiety disorder. They had, on average, two co-occurring mental state disorders and one co-occurring personality disorder, in addition to BPD. The three most commonly co-occurring personality disorders were antisocial (29%), avoidant (26%), paranoid (21%).

**Table 1:** Baseline demographic and clinical characteristics of participants who completed 12 month outcome measures

	Total sample (*N* = 98)	
	n (%)	
Female	78 (79.6)	
Age, *µ*(σ)	18.8 (2.7)	
Social disadvantage rank		
Low	17 (17.3)	
Medium	49 (50.0)	
High	31 (31.6)	
Stability of accommodation		
Low	5 (5.1)	
Medium	26 (26.5)	
High	66 (67.3)	
Caregiver completed secondary education	38 (38.8)	
Achieved age-appropriate secondary school	63 (64.3)	
Parent(s) born in Australia	40 (40.8)	
English as main language spoken at home	94 (95.9)	
Not living with a biological parent	64 (65.3)	
In a relationship	38 (38.8)	
Children	5 (5.1)	
Current mental state diagnoses		
Number, *µ*(σ)	2.5 (1.4)	
Any mood disorder	80 (81.6)	
Any anxiety disorder^a^	73 (74.5)	
Any eating disorder	4 (4.1)	
Any somatoform disorder	7 (7.1)	
Post-traumatic stress disorder	31 (31.6)	
Current personality disorder diagnoses		
Number, including BPD, *µ*(σ)	2.3 (1.4)	

*Note. NEET*, not in education, employment or training; *SOFAS* Social and Occupational Functioning Assessment Scale. *BPD* Borderline Personality Disorder; ^a^Excludes specific phobia and post-traumatic stress disorder

#### Hierarchical regression model 1: predictors of social adjustment at 12 months

The full model of demographic, symptom and behavioural and treatment characteristics to predict social adjustment at 12 months (SAS-SR) was statistically significant at p = 0.008, with a large effect size F2 = 0.412 (see Table 3). After adjusting for demographic variables, symptom and behavioural characteristics, having at least one caregiver in employment at baseline was the only variable to predict high levels of social adjustment at 12 months. Having at least one caregiver in employment was the strongest predictor, uniquely contributing 6.0% of the variance in social adjustment at 12 months (see Table 2).

#### Hierarchical regression model 2: predictors of interpersonal problems at 12 months

The full model of demographic, symptom and behavioural and treatment characteristics predicting interpersonal problems (IIP-C) at 12 months was statistically significant at p = 0.004 with a large effect size F2 = 0.522 (see Table 3). An assessment of coefficients showed that, after adjusting for demographic variables, symptom and behavioural characteristics, higher ratings of interpersonal functioning, lower ratings of BPD severity and a lower number of personality disorder diagnoses at baseline predicted fewer interpersonal problems at 12 months. BPD severity was the strongest predictor, uniquely contributing 8.0% of the variance, followed by baseline values of interpersonal function which uniquely contributed 7.0% of the variance, followed by the number of personality disorders at baseline which uniquely contributed 6.0% of the variance in interpersonal problems at 12 months (see Table 3).

**Table 2:** Model 1. Hierarchical multiple regression model predicting social adjustment from demographic, symptom and behavioural, and treatment characteristics

	Social Adjustment/Role Function							
	Block 1a		Block 1b		Block 1c		Block 1d	
Variables (at Baseline)	sr_i_^2^	β	sr_i_^2^	β	sr_i_^2^	β	sr_i_^2^	β
SAS-SR at Baseline	0.12	0.35***	0.12	0.36***	0.03	0.22	0.02	0.18
In relationship (Y/N)			0.06	-0.27*	0.05	-0.25*	0.03	-0.21
Social disadvantage status			0.02	0.15	0.04	0.21	0.04	0.21
Caregiver working (Y/N)			0.06	-0.25*	0.06	-0.26*	0.06	-0.25*
NEET (Y/N)			0.01	-0.10	0.01	-0.11	0.01	-0.13
Number of PD					0.01	-0.13	0.01	-0.13
BPDSI					0.01	0.15	0.01	0.15
SOFAS					0.02	-0.19	0.03	-0.21
Number of Medications					0.01	0.12	0.02	0.14
Working Alliance Inventory							0.01	-0.13
R^2^	0.12		0.29		0.33		0.34	
F	8.68***		4.70***		2.94**		2.77**	
ΔR^2^	0.12		0.17		0.04		0.01	
ΔF	8.68***		3.37**		0.82		1.14	

*Note. NEET* Not in education, employment or training; *PD* = Personality disorders; *BPDSI* Borderline Personality Disorder Severity index; *SOFAS* Social and Occupational Functioning Assessment Scale. *p < 0.05, **p < = 0.01, ***p < 0.005

**Table 3:** Model 2. Hierarchical multiple regression model predicting interpersonal problems from demographic, symptom and behavioural and treatment characteristics

	Interpersonal Problems							
	Block 2a		Block 2b		Block 2c		Block 2d	
Variables (at Baseline)	sr_i_^2^	β	sr_i_^2^	β	sr_i_^2^	β	sr_i_^2^	β
Interpersonal problems at Baseline	0.14	0.38**	0.14	0.39**	0.08	0.42*	0.07	0.41*
In relationship (Y/N)			0.06	-0.25	0.04	-0.24	0.04	-0.22
Highest education reached by caregiver			0.03	0.17	0.02	0.15	0.02	0.14
Number of MSD					0.05	0.29	0.05	0.30
Number of PD					0.06	-0.32*	0.06	-0.31*
BPDSI					0.08	0.38*	0.08	0.39*
MADRS					0.01	-0.10	0.01	-0.14
DERS					0.04	-0.31	0.04	-0.31
SOFAS					0.04	-0.25	0.04	-0.25
Number of substances					0.01	0.10	0.01	0.08
Number of medications					0.02	0.15	0.02	0.16
Working Alliance Inventory							0.01	-0.10
R^2^		0.14		0.21		0.43		0.44
F		9.22***		4.70**		3.20***		2.96***
ΔR^2^		0.14		0.07		0.23		0.01
ΔF		9.22***		2.24		2.30*		0.58

*Note. MSD* Mental State Disorder; *PD* Personality disorders; *BPD* Borderline Personality Disorder; *MADRS* Montgomery–Åsberg Depression Rating Scale; *BPD-SI* Borderline Personality Disorder Severity index; *DERS* Difficulties in Emotion Regulation Scale; *SOFAS* Social and Occupational Functioning Assessment Scale. *p < 0.05, **p < = 0.01, ***p < 0.005

## Discussion

This is the first study to investigate the baseline demographic, clinical, and treatment characteristics that predict higher levels of functioning at 12-month follow up among young people receiving early intervention for BPD. Four main findings emerged from this study. First, each of lower BPD severity, fewer co-occurring personality disorder diagnoses, and fewer interpersonal problems at baseline uniquely predicted fewer interpersonal problems at 12 months. Second, having at least one caregiver in employment at baseline uniquely predicted higher levels of social adjustment at 12 months. Third, suicide attempts (SA) and self-harm (SH) over the previous 12 months did not seem to be influential upon functional outcomes and were not included in the final model. Fourth, having at least one caregiver in employment at baseline uniquely predicted higher levels of social adjustment at 12 months.

The baseline characteristics that predict fewer interpersonal problems at 12 months (BPD severity, number of co-occurring PD diagnoses, fewer interpersonal problems) fall under the rubric of indicators of PD severity. The finding that lower BPD severity at baseline uniquely predicted fewer interpersonal problems following treatment is supported by previous epidemiological research showing that children and adolescents from the community with lower levels of BPD severity experienced improved peer relationships at 2-, 4-, and 20-year follow-up [16–18]. These findings are also supported by a cross-sectional study of adolescents (*N* = 177; aged 15–18 years) receiving outpatient treatment for BPD, which found that poorer peer relationships were predicted by a diagnosis of a PD (BPD or other PD), when adjusting for disruptive behaviour disorders, mood disorders, substance use disorders and anxiety disorders [36]. While BPD severity and interpersonal problems are overlapping constructs, their trajectories do not exhibit a 1:1 correspondence [37], with impairments in social functioning persisting at 2 [38], 10 [39] and 16 years [40] follow-up after the diagnostic criteria for BPD have attenuated. Moreover, the IIP-C measures a much broader range of interpersonal problems than the BPD criteria, which are limited to relationship instability and abandonment. This finding suggests that targeted intervention early in the course of this disorder [41], when young people might have lower cumulative burden of co-occurring PDs and interpersonal problems, might lead to fewer interpersonal problems later in life [42].

A higher level of social adjustment at 12 months was uniquely predicted by having at least one caregiver in employment. Parenthetically, it is noteworthy that social adjustment at 12 months was not predicted by factors that are commonly used as selection criteria for clinical service provision, i.e., indicators of clinical severity (e.g., BPD or depression severity, self-harm, suicide attempts), or the level of social adjustment at presentation. The main finding is supported by a large epidemiological study (*N* = 2281) in which a lower level of exposure to parental joblessness during childhood was associated with improved wage outcomes in early adulthood, even after adjusting for demographic and socioeconomic factors [43]. Although these findings are associations, two mechanisms might explain how caregiver employment might predict long-term occupational functioning in their offspring. First, the ‘resource-based framework’ [44] suggests that parental employment increases a caregiver’s ability to gain access to resources that might be beneficial to the child’s development, including food, housing, and the capacity to invest in early educational environments conducive to growth and learning. In turn, this might enhance the formation of skills required for future labour force attachment [45]. Second, the ‘socialisation framework’ [46] suggests that the presence of work role models and positive parental attitudes toward employment might increase a child’s self-efficacy and motivation for employment [47]. The current findings highlight the importance of further research designed to investigate the potential roles of caregiver educational and occupational attainment in treatment outcome. Such research might investigate the importance of including caregivers in early intervention programs for young people with BPD, or the effectiveness of interventions directed toward caregivers that might enhance educational and occupational attainment (or other outcomes) for caregivers and/or young people.

The finding that SA and SH over the previous 12 months did not seem to be influential in 12-month functional outcomes reinforces the case to reconsider the influence of these factors upon decisions about clinical care and clinical trials. In many clinical settings, recent SA and SH garner much of the short-term focus of care and are highly influential in clinical decision-making, especially with regard to offering treatment or, paradoxically, excluding participants from clinical trials [48, 49]. Evidence supports SA and SH as a population level transdiagnostic marker of psychopathology and suicide risk among young people [49]. However, the current finding adds weight to the argument that, among clinical populations, where SA and SH are ubiquitous [99% lifetime history among the MOBY sample; 50], and have limited utility as a test for later suicide because of their modest sensitivity and low positive predictive value [51, 52], SA and NSSI should not exert disproportionate influence upon who is selected for treatment or excluded from research.

### Strengths and limitations

This is the first study to investigate predictors of functional outcomes among young people receiving early intervention for BPD. Strengths of this study include the intentional recruitment of a ‘real-world’ sample of young people, typical of frontline clinical services, who, prior to involvement in this study, had never received a diagnosis of or specialised treatment for BPD. Also, the methodological process of eliminating variables prior to the final regression analysis, along with the relatively large sample size, meant that a large number of predictor variables could be examined, without compromising the power of the study. Despite the large number of variables measured, there remains a substantial amount of unexplained variance, suggesting that there might be important variables not usually measured in clinical trials for personality disorder that might influence functional outcomes. The substantial unexplained variance coupled with this study’s cross-sectional design precludes causal inference. Large-scale longitudinal investigations are necessary to establish the temporal sequence between the predictor and outcome variables.

Limitations include that the method for defining social disadvantage rank was based upon postcode, rather than individual or household variables. It is possible that the relationship between having at least one caregiver in employment and better functional outcomes might be explained by other individual or household indicators of social disadvantage. It is also possible that participants with poor outcomes are under-represented at the 12-month time point, as the presence and number of mental disorders at follow-up, rather than baseline, has been found to be significantly associated with follow-up contact difficulty in longitudinal studies among young people [53]. While personality functioning, as defined in the DSM-5 Alternative Model for Personality Disorders (AMPD), was not measured in this study (which was commenced prior to DSM-5 and validated measures of personality functioning), evidence suggests that ‘severe’ PD and BPD are largely synonymous [54]. Therefore, the current sample is likely to represent individuals at the most severe end of the personality functioning spectrum. Consequently, these findings might have limited generalisability to young people with less severe personality pathology and less socio-economic disadvantage.

## Conclusions

There were few baseline predictors of outcome identified in this clinical trial of early intervention for young people with first-presentation BPD. Of those that were identified, perhaps unsurprisingly, young people who presented with indicators of lower BPD severity were more likely to have fewer interpersonal problems at 12-month outcome. The finding in regard to caregiver employment and better social adjustment suggests that future research should focus upon individual or household indicators of social disadvantage as moderators of outcome in early intervention for BPD. Finally, the lack of support for commonly used selection criteria for clinical service provision (e.g., SA, SH, depression severity, substance use) as predictors of functional outcome suggests the need for a reconsideration of weight given to these criteria in treatment selection or exclusion from clinical trials.

## Electronic supplementary material

Below is the link to the electronic supplementary material.

Supplementary Material 1

## Data Availability

Data supporting results is available upon request to the corresponding author.
