# Supplementary material for: Baseline correlates of functional impairment at 12 months in young people with borderline personality disorder: findings from the MOBY trial
Source: Eur Child Adolesc Psychiatry. 2025 May 28;34(11):3427–35. doi: 10.1007/s00787-025-02742-5 (PMC12647327; doi:10.1007/s00787-025-02742-5)
Supplement: Supplementary file 1 — Supplementary Material 1 [file 787_2025_2742_MOESM1_ESM.docx]

Supplementary Table 1. Demographic, symptom and treatment variables examined and eliminated prior to regression model

|  | Variables assessed for inclusion in model | Model 1 – IIP-C as DV^a^ | Model 2 – SAS-SR as DV^a^ |  |
| --- | --- | --- | --- | --- |
| Demographics | 1. Sex 2. Age 3. Social disadvantage rank 4. Stability of accommodation (High, Med, Low) 5. Relationship status (Y/N) 6. Caregiver working (Y/N) 7. Highest education reached by caregiver 8. NEET status 9. No. of children 10. Language spoken at home 11. Parents born overseas^b^ | 1. Relationship       status (Y/N)   1. Highest education reached by caregiver | 1. Social disadvantage rank  2. Relationship status (Y/N)  3. A caregiver     working (Y/N)  4.  NEET status | |
| Symptom and behaviours | 1. No. of MSD 2. No. of PD 3. Inclusion of MSD and PD variables separately (including PTSD) 4. BPDSI total 5. MADRS total 6. DERS total 7. No. of medications 8. No. of medication classes 9. Frequency of suicide and non-suicidal self-injury 10. Age of onset of self-harm^c^ 11. No. of substances 12. SOFAS | 3. No. of MSD  4. No. of PD  5. BPDSI total  6. MADRS total  7. DERS total  8. No. of      medications  9. No. of substances used  10. SOFAS | 5. No. of PD  6. BPDSI total  7. No. of      medications  8. SOFAS | |
| Treatment | 24. No. of contacts with mental health services^d^  25. No. of emergency visits or inpatient admissions.  26. Average Client Satisfaction  27. Average Working Alliance Score from client^e^ | 11.Average Working Alliance Inventory | 8. Average Working Alliance Inventory |  |

Note: NEET = Not in Education or Employment; MSD = Mental State Disorder; PD = Personality disorders; MADRS = Montgomery–Åsberg Depression Rating Scale; BPD-SI = Borderline Personality Disorder Severity index; DERS = Difficulties in Emotion Regulation Scale; SOFAS = Social and Occupational Functioning Assessment Scale.

^a^Final predictors included in model after elimination of variables according to elimination criteria outlined in statistical analyses

^b^Parents born overseas - assessed both as a dichotomous variable and interval variable (interval: no parents, one parent, two parents)

^c^Based on frequency of suicide attempts and acts of self-harm a year prior to baseline [[Suicide attempt self-injury interview; 47]](https://paperpile.com/c/FglH3b/u7wPC/?prefix=Suicide%20attempt%20self-injury%20interview%3B);

^d^Contacts might include treatment sessions, case management, psychiatry/medication reviews and family work sessions.

^e^A 27- item questionnaire which assesses the level of working alliance between the client and therapist, measured on a 7 point scale [[1= never, 7 = always; 48]](https://paperpile.com/c/FglH3b/Seg4z/?prefix=1%3D%20never%2C%207%20%3D%20always%3B%20). Average client ratings taken across all time points (baseline, 6 months and end of treatment).
